# Outbreak of Human Pneumonic Plague with Dog-to-Human and Possible Human-to-Human Transmission — Colorado, June–July 2014

**Published:** 2015-05-01

**Authors:** Janine K. Runfola, Jennifer House, Lisa Miller, Leah Colton, Donna Hite, Alex Hawley, Paul Mead, Martin Schriefer, Jeannine Petersen, Colleen Casaceli, Kristine M. Erlandson, Clayton Foster, Kristy L. Pabilonia, Gary Mason, John M. Douglas

**Affiliations:** 1 Tri-County Health Department, Colorado; 2 Colorado Department of Public Health and Environment; 3 Division of Vector-Borne Diseases, National Center for Emerging and Zoonotic Infectious Diseases, CDC; 4 Platte Valley Medical Center, Colorado; 5 University of Colorado Anschutz Medical Campus; 6 Colorado State University Veterinary Diagnostic Laboratories

On July 8, 2014, the Colorado Department of Public Health and Environment (CDPHE) laboratory identified *Yersinia pestis*, the bacterium that causes plague, in a blood specimen collected from a man (patient A) hospitalized with pneumonia. The organism had been previously misidentified as *Pseudomonas luteola* by an automated system in the hospital laboratory. An investigation led by Tri-County Health Department (TCHD) revealed that patient A’s dog had died recently with hemoptysis. Three other persons who had contact with the dog, one of whom also had contact with patient A, were ill with fever and respiratory symptoms, including two with radiographic evidence of pneumonia. Specimens from the dog and all three human contacts yielded evidence of acute *Y. pestis* infection. One of the pneumonia cases might have resulted through human-to-human transmission from patient A, which would be the first such event reported in the United States since 1924. This outbreak highlights 1) the need to consider plague in the differential diagnosis of ill domestic animals, including dogs, in areas where plague is endemic; 2) the limitations of automated diagnostic systems for identifying rare bacteria such as *Y. pestis;* and 3) the potential for milder plague illness in patients taking antimicrobial agents. Hospital laboratorians should be aware of the limitations of automated identification systems, and clinicians should suspect plague in patients with clinically compatible symptoms from whom *P. luteola* is isolated.

## Investigation and Results

Patient A, a previously healthy middle-aged man, developed fever and cough on June 28. Over the next 24 hours his condition worsened with increasing cough and the production of bloody sputum. He was admitted to a local hospital where he was diagnosed with pneumonia (Figure). Blood cultures collected on June 30 grew a gram-negative rod that was initially identified as *P. luteola* using an automated identification system. Over the next 6 days patient A’s respiratory status deteriorated, and he was transferred to another facility where he required intubation. Because of the severity of his illness and previous reports of misidentification of *Y. pestis* as *P. luteola* (1,2), the isolate was sent to the CDPHE laboratory for further testing. On July 8 the specimen was correctly identified as *Y. pestis*, and patient A received a diagnosis of pneumonic plague. Patient A was treated with broad-spectrum antibiotics, including levofloxacin and streptomycin, and recovered after hospitalization for 23 days.

TCHD initiated an investigation, consisting of interviews with patient A’s family, evaluation of potential exposures to the patient, and an environmental assessment to determine the risk for further disease transmission. The investigation revealed that patient A’s dog, a male American pit bull terrier aged 2 years, became ill with fever, jaw rigidity, drooling, and right forelimb ataxia on June 24 (Table). The dog was kept overnight at a veterinary clinic and humanely euthanized the following day after developing dyspnea and bloody sputum. Patient A had close contact with the dog during euthanasia. Necropsy revealed gastric and pulmonary hemorrhage. Samples tested negative for evidence of rabies virus infection and anticoagulants; histopathologic examination of the tissues was declined by patient A. Following patient A’s diagnosis with plague, liver and lung tissues from the dog were tested for *Y. pestis*, and results were positive by both polymerase chain reaction assay and culture. Archived formalin-fixed tissues from the dog were processed for histopathology, revealing severe acute bronchopneumonia with intra-alveolar bacteria. The investigation also identified three other persons who had been in close contact with the ill dog, one of whom who also had contact with patient A. All three subsequently received diagnoses of plague, and all three recovered (Table, Figure).

On June 30, 2 days after patient A became ill, patient B, a female veterinary clinic employee, developed a fever and cough and visited an urgent care facility, where bronchitis was diagnosed. She reported close contact with the ill dog on June 24–25. After her symptoms failed to improve with self-initiated amoxicillin/clavulanic acid, patient B visited an emergency department on July 5, received a diagnosis of pneumonia, and was treated with azithromycin, with improvement over the next several days. After notification on July 10 of her exposure to plague, she visited a health care provider and was treated with oral levofloxacin. A polymerase chain reaction test on a sputum specimen was positive for *Y. pestis*. Subsequent testing of paired acute and convalescent serum specimens demonstrated a fourfold increase in antibody titers to *Y. pestis*, indicative of recent infection (Table).

Patient C, a female veterinary clinic employee, also had close contact with the dog on June 24–25 and self-initiated a 6-day course of oral doxycycline on June 25. On July 4, she experienced fever, chills, myalgia, and fatigue; symptoms progressed to chest tightness and cough. Following notification of the exposure to plague on July 9, patient C self-initiated a second course of doxycycline and was medically evaluated later that day. Crackles were heard during chest auscultation; however, results of a chest radiograph were normal. A full course of oral doxycycline was continued with resolution of symptoms. Initial and follow-up serum specimens tested positive for antibody to *Y. pestis*, with a greater than fourfold decrease in antibody titers at follow-up 6 months later (Table).

On July 4, patient D, a woman who was a close contact of patient A, experienced chest tightness, dyspnea, and fever. She was evaluated at an emergency department, received a diagnosis of pneumonia, and was treated with oral levofloxacin. Patient D handled the body of the dog on June 25 after it died, at one point getting blood on her hands. She also had extended close contact with patient A on June 29–30 while he was coughing bloody sputum. On July 8, after patient A was identified with pneumonic plague, patient D was hospitalized and treated with levofloxacin and streptomycin. Paired acute and convalescent serum specimens for patient D demonstrated a greater than fourfold increase in antibody titers to *Y. pestis* (Table).

## Public Health Response

TCHD evaluated potential exposures from each patient and conducted an environmental assessment to determine the risk for further disease transmission. Case status was assigned according to case definitions developed by the Council of State and Territorial Epidemiologists for CDC’s National Notifiable Diseases Surveillance System.*

Medical personnel and personal contacts of all four patients were notified of their possible exposure to plague. A total of 114 persons had close contact with the dog or one or more of the human patients: 36 in veterinary settings, 58 in human health care settings, and 20 as close personal contacts. Antimicrobial prophylaxis was recommended for 88 persons interviewed within 7 days of exposure. The remaining 26 were advised to monitor for fever for 7 days and to seek medical attention immediately if symptoms occurred.

On July 9, TCHD surveyed patient A’s property for evidence of plague. Live rabbits were observed on the property but no other wildlife. Inactive prairie dog burrows were present; however, it was reported that the prairie dog colony had been intentionally eradicated in October 2013.

CDPHE issued press releases for public awareness and Health Alert Network notifications to health care providers and veterinarians on July 9, 10, and 18. Medical facilities were instructed to use droplet precautions for persons with suspected plague. TCHD staff members distributed information on plague symptoms and transmission risk to homes in the vicinity of the index patient. No further cases have been identified.

### Discussion

Plague is a rare but life-threatening zoonosis caused by *Y. pestis.* A median of eight cases of human plague are reported annually in the United States (3), primarily among residents of semirural areas in New Mexico, Arizona, Colorado, and California. Normally a pathogen of rodents, *Y. pestis* is transmitted to humans through the bite of infected rodent fleas or direct contact with the tissues or secretions of infected animals. Bubonic plague, characterized by fever and painful regional lymphadenopathy, results from percutaneous exposure and accounts for approximately 85% of reported cases. Pneumonic plague occurs as either a complication of untreated bubonic plague (10%–13% of all cases) or as a primary pneumonia following inhalation of infectious droplets (2% of all cases) (4). Untreated pneumonic plague has a fatality rate of ≥93% and can be spread from person to person through aerosols generated during coughing. A third clinical form, septicemic plague, is characterized by fever and shock without localizing signs or symptoms. Laboratory diagnosis of plague is based on culture or polymerase chain reaction assays of blood, sputum, or lymph node aspirates, or on serology. Effective therapy includes aminoglycosides and doxycycline. In addition, the fluoroquinolones levofloxacin and ciprofloxacin have been approved recently by the Food and Drug Administration based on animal studies.† The advent of antimicrobial therapy has reduced overall plague mortality from >60% to approximately 16% (3,5,6).

In this outbreak, all four patients had laboratory-confirmed plague, including three patients (A, B, and D) with clinical and radiographic evidence of pneumonia. The fourth patient (C) had an atypical presentation with respiratory symptoms but no radiographic evidence of pneumonia, possibly as a result of partial treatment immediately after exposure. Three patients (A, B and C) became ill shortly after exposure to an ill infected dog. The source of infection for patient D is less certain because she had exposure to both the dog on June 25–26 (an incubation period of 9–10 days) and to patient A on June 29–30 while he had hemoptysis (an incubation period of 5–6 days). The shorter incubation period is more typical of plague and therefore supports human-to-human transmission (6). Nevertheless, transmission from the dog cannot be excluded given the animal’s role in the other three infections and because incubation periods of up to 10 days have been reported, although rarely (7). Primary pneumonic plague is rare in the United States with only 74 cases reported during 1900–2012, and this event represents the largest outbreak and the first instance of possible human-to-human transmission since an outbreak in Los Angeles in 1924 (3,5).

*Y. pestis* infection in dogs generally is either asymptomatic or the cause of only a mild, self-limiting febrile illness (8). Dogs can play a role in human infection through transport of rodent fleas into the home (8,9). This outbreak began with illness in a pet dog, a previously unrecognized source of plague exposure in the United States. The only previously published case of direct transmission of plague from a dog to a human was reported from China in 2009 (10). Although symptomatic plague in dogs is rare, veterinarians should consider the possibility of *Y. pestis* infection in ill dogs with wildlife exposure in areas where plague is endemic.

This outbreak is notable for the several factors that delayed its recognition. First, patient A’s bacterial isolate initially was identified as *P. luteola* by an automated blood culture system, and the correct identification of *Y. pestis* was only made 7 days later. This delay resulted in the exposure of numerous medical personnel. Misidentification and a resulting delayed diagnosis have been previously reported, reinforcing the need for critical evaluation of results from automated systems and education of hospital microbiologists regarding this limitation (1,2). Among 12 *Y. pestis* isolates obtained from U.S. patients during 2010–2013, at least three (25%) were originally misidentified by automated systems (Division of Vector-borne Diseases, National Center for Emerging and Zoonotic Infectious Diseases, CDC, unpublished data, 2015). Second, the spectrum of disease was broader than usual for pneumonic plague (7), with two of the four patients not requiring hospitalization. The clinical course of the milder cases might have been modified by self-administration of antibiotics or medical prescription of azithromycin, an antibiotic not recommended for plague. Pneumonia is the only form of plague with the potential for human-to-human transmission. Delayed recognition because of inaccurate laboratory test results and atypical clinical presentations can lead to high numbers of potential exposures to health care workers, laboratory workers, and other close contacts.

What is already known on this topic?

Rapid identification of plague is critical in patients who live in, or who have recently traveled to, regions where plague is endemic, including the western United States. The three most common forms of plague are bubonic, pneumonic, and septicemic, with the majority of cases presenting as bubonic. Although the rarest form of plague (approximately 2% of reported cases), primary pneumonic plague has a high (≥93%) mortality rate when left untreated.

What is added by this report?

The outbreak in Colorado represents the largest outbreak of pneumonic plague in the United States since 1924. The source of the outbreak was a dog with pneumonic plague, an atypical occurrence because dogs infected with *Yersinia pestis* generally are either asymptomatic or exhibit mild self-limiting febrile illness and are not considered a direct source of human infection. Four persons developed plague after exposure to the ill dog; one of the patients also had close contact with the index patient after he developed plague pneumonia, supporting possible human-to-human transmission. Diagnosis in the index case was delayed because of misidentification of a bacterial isolate as *Pseudomonas luteola* by an automated blood culture system. The spectrum of disease in this outbreak was broader than usual for pneumonic plague, with two of the four patients not requiring hospitalization, possibly as a result of self-administration of antibiotics or medical prescription of azithromycin, an antibiotic not recommended for plague.

What are the implications for public health practice?

Plague should be considered in the differential diagnosis of dogs with respiratory illness in areas where plague is endemic. The results of automated blood culture systems should be evaluated critically when rare diseases are suspected. Patients with suspected pneumonic plague should be isolated before laboratory confirmation and treated with appropriate antibiotics. Blood or sputum cultures should be sent to state public health laboratories for confirmation.

Although human plague is rare in North America, it remains a public health concern in the western United States where *Y. pestis* circulates among wild rodent populations. The risk for plague can be minimized by avoidance of possibly infected rodents (e.g., prairie dogs) and their fleas. All suspected or confirmed plague cases and rodent die-offs in areas where plague is endemic should be reported immediately to public health officials so that exposures can be minimized to prevent additional transmission. Once plague is suspected, appropriate precautions and treatment should be initiated immediately, and clinical specimens should be collected and tested as soon as possible. Early recognition of plague, especially the pneumonic form, is critical to effective clinical management and a timely public health response. Veterinarians should consider plague in the differential diagnosis of ill domestic animals, including dogs, in areas where plague is endemic.

## Figures and Tables

**FIGURE:**
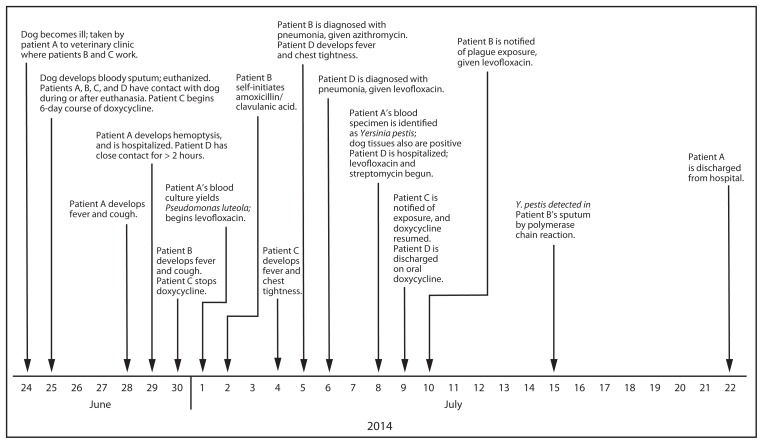
Timeline of diagnoses and treatment for patients identified in a pneumonic plague outbreak — Colorado, 2014

**TABLE:** Dates of exposure and illness onset and test results for patients identified in a pneumonic plague outbreak — Colorado, June–July 2014

Patient	Date of exposure (source)	Onset of illness	Chest radiograph findings	Hospitalized	Laboratory test results										

					Polymerase chain reaction			Culture			Serologic testing				

											Initial			Follow-up	
			
					Specimen	Date	+/−	Specimen	Date	+/−	Specimen	Date	Titer	Date	Titer
Dog	Unknown	June 24	PNA	Yes	Liver/lung tissue	June 26	+	Liver/lung tissue	June 26	+	NT	NT	NT	NT	NT
A	June 25	June 28	PNA	Yes	Blood	June 29	+	Blood	June 29	+	NT	NT	NT	NT	NT
B	June 25	June 30	PNA	No	Sputum	July 10	+	Sputum, blood	July 10	−	Blood	July 10	1:64	July 24	1:64
														Jan 12 2015	1:256
C	June 25	July 4	no evidence of PNA	No	Blood	July 9	−	Blood	July 9	−	Blood	July 9	1:32	July 24	1:32
														Jan 12 2015	−
D	June 25 (dog)	July 5	PNA	Yes	Blood	July 6	−	Blood	July 6	−	Blood	July 6	−	July 12	1:32
	June 29 (patient A)													July 23	1:32

**Abbreviations:** PNA = pneumonia; + = positive test result; − = negative test result; NT = not tested.

## Footnotes

* Available at http://wwwn.cdc.gov/NNDSS/script/casedef.aspx?CondYrID=800&DatePub=1/1/1996.

† Available at http://www.fda.gov/downloads/Drugs/DrugSafety/ucm088619.pdf (levofloxacin) and http://www.fda.gov/downloads/Drugs/DrugSafety/ucm246794.pdf (ciprofloxacin).
